# Sex differences in the management of persons with dementia following a subnational primary care policy intervention

**DOI:** 10.1186/s12939-020-01285-2

**Published:** 2020-10-06

**Authors:** Nadia Sourial, Geneviève Arsenault-Lapierre, Eva Margo-Dermer, Mary Henein, Isabelle Vedel

**Affiliations:** 1 Département de Médecine de Famille et de Médecine d’Urgence, Faculté de médecine, Université de Montréal, Montreal, Canada; 2 Centre de Recherche du Centre Hospitalier de l’Université de Montréal, Université de Montréal, Montreal, Canada; 3 Lady Davis Institute for Medical Research, Jewish General Hospital, Montreal, Canada; 4 Department of Family Medicine, McGill University, Montreal, Canada

**Keywords:** Sex, Gender, Equity, Health policy, Evaluation

## Abstract

**Background:**

The influence of sex and gender on the risk of dementia, its clinical presentation and progression is increasingly being recognized. However, current dementia strategies have not explicitly considered sex and gender differences in the management of dementia to ensure equitable care. The objective of this study was to examine the moderating effect of sex on the quality of care following the implementation of the Quebec Alzheimer Plan (QAP).

**Methods:**

We conducted a secondary analysis of the evaluation of the QAP consisting of a retrospective chart review of 945 independent, randomly-selected patient charts of males and females 75+ years old with dementia and a visit to one of 13 participating Family Medicine Groups before (October 2011–July 2013) and after (October 2014 – July 2015). The quality of dementia care score, based on Canadian and international recommendations and consensus guidelines, consisted of documented assessments in 10 domains. We used a mixed linear regression model to measure the interaction between sex and the implementation of the QAP on the quality of dementia care score, adjusting for age and number of medications.

**Results:**

We found that improvements in the quality of dementia care following the QAP were larger for men than women (mean difference = 4.97; 95%CI: 0.08, 9.85). We found that men had a larger improvement in four indicators (driving assessments, dementia medication management, Alzheimer Society referrals, and functional status evaluation), while women had a smaller improvement in three (home care needs, behavioural and psychological symptoms of dementia, and weight). Men were prescribed fewer anticholinergics post-QAP, while women were prescribed more. Cognitive testing improved in men but decreased for women following the QAP; the opposite was observed for caregiver needs.

**Conclusion:**

While the overall quality of care improved after the implementation of the QAP, this study reveals differences in dementia management between men and women. While we identified areas of inequalities in the care received, it is unclear whether this represents inequities in access to care and health outcomes. Future research should focus on better understanding sex and gender-specific needs in dementia to bridge this gap and better inform dementia strategies.

## Background

Sex and gender are recognized as major drivers of health inequities [1–5]. For example, differences in health seeking behavior, access to informal caregivers, financial and social resources among men and women have been shown to create health disparities in access to care and health outcomes, especially affecting older women [6–9]. As such, the importance of integrating sex and gender in health research to prevent inequities in care is increasingly being recognized for sound evidence-based practice and policy [10, 11]. In particular, consideration of these issues in the development and implementation of health interventions has been advocated to ensure interventions are equitable and adapted to meet the needs of both men and women [12].

The influence of sex and gender is particularly relevant in dementia care. Evidence is emerging on the influence of sex and gender on the risk of dementia, its clinical presentation and progression [13]. Dementia disproportionately affects women, who account for 2 in 3 cases [13]. This higher prevalence has not only been attributed to women living longer on average than men but also due to underlying differences in disease and/or sex and gender-related risk factors [13–15]. For example, older women are more likely to have a lower educational attainment and engage in less exercise, both risk factors for dementia, due in part to historically greater parental roles in women than in men [16]. Women, who carry a greater caregiving burden, also have twice the risk of developing depression, a major risk factor for dementia, compared to men [16, 17].

In terms of the clinical presentation and progression of dementia, women tend to perform better on cognitive tests than men due to improved reserves in verbal memory [18]. As a result, women tend to be diagnosed later in the disease, leading to delayed management and more rapid decline after diagnosis than men [13, 18–20]. On the other hand, men with dementia have been shown to have a higher prevalence of severe comorbidities [21–24], and a higher likelihood to develop aggressive behavioral and psychological symptoms of dementia [25, 26]. Finally, as seen in the general older population, gender differences have been shown in men and women with dementia in terms of access to care, financial and social support [13, 14].

These sex and gender differences in dementia may require different approaches to the management of dementia. However, the role of sex and gender has been largely neglected in national and subnational dementia strategies seeking to improve the quality of dementia care through better detection, diagnosis and management of persons with dementia [27]. Only the most recent dementia strategies and plans consider differences in risk and health disparities based on sex and gender in their action plan but none explicitly discuss how sex and gender should be considered in the management of dementia to ensure equitable care [27, 28].

One subnational dementia care intervention, the Quebec Alzheimer Plan [29], aimed at improving dementia care in the primary care setting, was recently evaluated. The Quebec Alzheimer Plan was initiated in 2014 and is being implemented in Family Medicine Groups, primary care interdisciplinary clinics, across Quebec, Canada. It aims to enable and empower primary care clinicians to detect, diagnose, treat and follow-up patients living with dementia and their caregivers, while allowing for local adaptations, by providing financial support and training. The pilot phase of its implementation was previously evaluated and found to improve the quality of dementia care within primary care [30]. Further details on the intervention and early evaluation are available elsewhere [30, 31]. However, whether changes in the quality of dementia care were similar in both men and women have yet to be explored. Understanding potential sex differences is essential to adapt such ongoing strategies to ensure equity in their implementation and scale-up. The objective of this study was therefore to examine the moderating effect of sex on the impact of the Quebec Alzheimer Plan (QAP) on the quality of dementia care.

## Methods

### Setting, design and population

This study is a secondary analysis of the evaluation of the QAP which consisted of training and funding for primary care clinics in Quebec, Canada’s second largest province accounting for one-quarter of the population [32], to improve the quality of care of persons with dementia. Implementation of the QAP took place between 2014 and 2015. The evaluation was conducted through a retrospective chart review of 945 independent, randomly-selected patient charts of males and females 75 years old and older with dementia (including mild cognitive impairment) and with a visit to one of 13 participating clinics before (pre-period: October 2011–July 2013) and after (post-period: October 2014 – July 2015) the implementation of the QAP [30]. Further details are available elsewhere [30].

### Study variables

Information on age, sex, living arrangement, the number of medications, the prevalence of antipsychotic use, and the type of dementia were collected from patient charts.

The study outcome was the quality of dementia care as measured by a score based on Canadian and international recommendations and consensus guidelines for the care of persons with dementia [29, 33–35]. This score consisted of documented assessments in the following 10 domains: cognitive testing, evaluation of functional status, behavioral and psychological symptoms of dementia (BPSD), weight, caregiver needs, driving status, home care needs, community service needs (e.g. Alzheimer Society), absence of anticholinergic medication, and management of dementia medications. The score was calculated by summing the number of indicators performed divided by the number of eligible indicators for each patient. Patients’ eligibility for each indicator (for example a patient who is no longer driving would not need a driving evaluation) was assessed over the study period.

### Analysis

Patient characteristics before and after the QAP were described stratified by sex. A mixed-effect linear regression model was used to model the interaction between sex and the implementation of the QAP on the quality of dementia care score, adjusting for age and number of medications. A sensitivity analysis further adjusting for living arrangement was also conducted. As patients within clinics were likely to be more similar than patients across clinics, an identifier for the clinic was included as a random effect in the model to account for the effect of clustering on the variability in the data. Adjusted means and associated 95% confidence intervals were derived from the model. A bar graph was used to illustrate the frequency of each of the 10 quality of care indicators for both men and women and in both study periods.

## Results

Differences in the characteristics of men and women were comparable in the pre and post period (Table 1). Women accounted for a majority of the sample, 63% in the pre-period, 61% in the post-period. In each period, women were on average 1 year older than men and were taking one additional medication than men. We observed more cases of Alzheimer’s disease in women, while men had a higher prevalence of mixed dementia. A greater proportion of women in our study lived alone or with someone else other than a spouse or child than men (Table 1).

**Table 1:** Patient characteristics

	Pre-QAP^a^ Implementation *N* = 455		Post-QAP Implementation *N* = 490	
	Men *n* = 169 (37%)	Women *n* = 286 (63%)	Men *n* = 190 (39%)	Women *n* = 300 (61%)
Age in years, median (min-max)	83.1 (75.0–99.5)	84.3 (75.0–102.0)	82.8 (75.1–98.3)	83.6 (75.2–100.9)
Number of medications, median (min-max)	11.0 (0.0–37.0)	12.0 (1.0–38.0)	12.0 (0.0–44.0)	13.5 (0.0–34.0)
Antipsychotic use, (%)	43 (25.4)	74 (25.9)	38 (20.0)	60 (20.0)
Diagnosis, number (%)				
Alzheimer’s Disease	37 (23.2)	80 (29.3)	50 (28.4)	91 (31.1)
Mixed Dementia	39 (24.5)	49 (17.9)	25 (14.2)	33 (11.3)
Vascular Dementia	8 (5.0)	10 (3.7)	7 (4.0)	10 (3.4)
Lewy Body Dementia	6 (3.8)	6 (2.2)	2 (1.1)	3 (1.0)
Other Dementias	2 (1.2)	5 (1.8)	1 (0.6)	2 (0.6)
Unspecified Dementia	18 (11.3)	44 (16.1)	28 (15.9)	47 (16.0)
Mild Cognitive Impairment	26 (16.4)	44 (16.1)	32 (18.2)	52 (17.7)
Unspecified Cognitive Impairment	23 (15.4)	36 (14.2)	31 (17.6)	55 (18.8)
Living Arrangement, number (%)				
Alone	16 (9.5)	48 (16.8)	25 (13.2)	58 (19.3)
Spouse and/or Child	105 (62.1)	102 (35.7)	120 (63.2)	125 (41.7)
Other	34 (20.1)	102 (35.7)	34 (17.9)	93 (31.0)
Unknown	14 (8.3)	34 (11.9)	11 (5.8)	24 (8.0)

Legend: ^a^*QAP* Quebec Alzheimer Plan

**Fig. 1:**
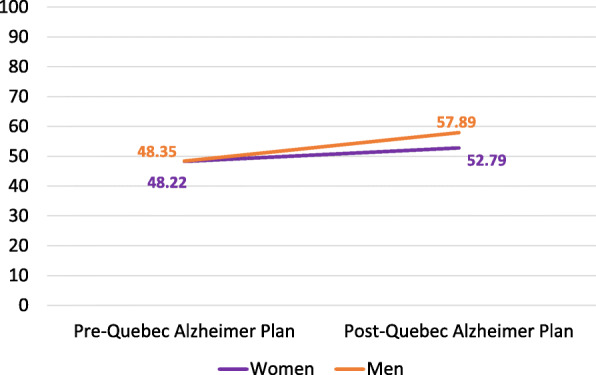
Quality of follow-up scores for men and women with neurocognitive disorders before and after implementation of the Quebec Alzheimer Plan (QAP). Footnote: The interaction between sex and the implementation of the QAP was found to be statistically significant (mean difference: 4.97, 95% CI: 0.08, 9.85; see Table 2)

Figure 1 presents the adjusted mean quality of dementia care score for men and women, pre and post-QAP implementation. While the score in the pre-period was similar for men and women (48.4 vs. 48.2), a greater increase in the score was observed in men than in women in the post period (57.9 vs. 52.8). This interaction between sex and the implementation of the QAP was found to be statistically significant (mean difference: 4.97, 95% CI: 0.08, 9.85; Table 2). The sensitivity analysis showed a similar interaction estimate between sex and the implementation of the QAP when accounting for living arrangement (mean difference 5.18; 95% CI: 0.28–10.08).

**Table 2:** Differential impact of the Quebec Alzheimer Plan for men and women with neurocognitive disorders

	Mean Difference (95% Confidence Interval)
Intercept	52.95 (32.77, 73.12)
Men^a^	0.04 (−3.49, 3.57)
Post-QAP^b,c^	4.59 (0.72, 8.46)
Men^*^Post-QAP	4.97 (0.08, 9.85)
Age	−0.05 (−0.28, 0.18)
Number of Medications	−0.03 (− 0.21, 0.14)

^a^Reference category is women

^b^Reference category is pre-QAP

^c^*QAP* Quebec Alzheimer Plan

**Fig. 2:**
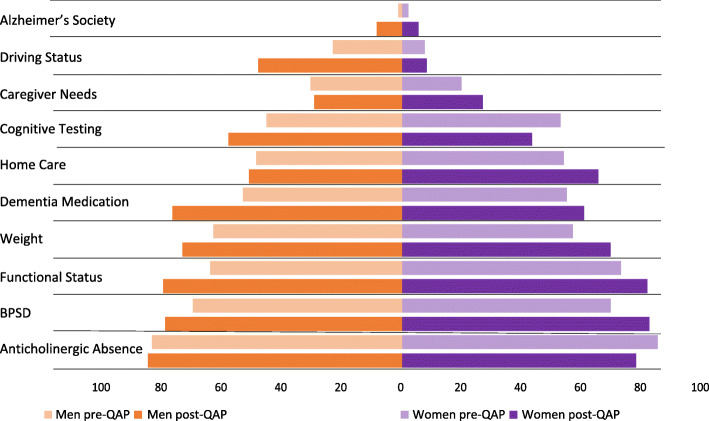
Quality of follow-up indicators assessed for men and women with neurocognitive disorders before and after implementation of the Quebec Alzheimer Plan (QAP). Footnote: All indicators refer to evaluation or assessments reported in the patient charts. Anticholinergic absence refers to the absence of anticholinergic medications in the patients list of medications. BPSD stands for Behavioural and psychological symptoms of dementia

Figure 2 presents the frequency of assessments of the 10 quality of dementia care indicators in men and women before and after the QAP. Men showed greater improvement in terms of Alzheimer Society referrals, driving assessments, dementia’s medication management and functional status evaluations. Home care needs, weight, and BPSD assessments increased by a greater margin in women. Frequency of cognitive testing increased in men following implementation of the QAP whereas testing decreased in women. Similarly, men were less often prescribed anticholinergics following the QAP than women. While caregiver needs assessments improved for women but decreased in men, the frequency of assessment was still higher in men followed the QAP.

## Conclusion

To our knowledge, this study is the first to assess sex differences in the impact of a population-level dementia plan. We found that improvements in the quality of dementia care following the QAP were larger for men than for women. Among the 10 quality of care indicators assessed, we found that men had a larger improvement in five of these indicators while women improved more than men in three indicators. Changes following the QAP in two indicators were in opposite directions for men and women. This study reveals differences in quality of dementia care between men and women following a subnational dementia policy. The results may suggest inequalities of care for women compared with men in dementia strategies.

Evidence with which to compare our results were extremely scarce. We found direct comparative literature in only two indicators: absence of anticholinergic medication and assessment of functional status. Our finding that men had less frequent use of anticholinergic medication was consistent with two studies [36, 37], but inconsistent with others which found no significant difference in anticholinergic use between men and women with dementia [38, 39]. In terms of functional status assessments, our study was consistent with one other study [40], which found hospitalized older women had more assessments than older men (9.1% of men did not have an assessment recorded, compared to 8.0% of women).

For the remaining indicators, only broader literature was found. We found that male drivers were more frequently assessed for driving than female drivers and that assessments of driving ability improved after QAP in men but not in women. This finding may be related to gender-social roles where older drivers have been found to be more frequently men [41]. Moreover, it has been shown that women are more likely to self-regulate and stop driving than men [42]. Together, these differences may have influenced clinicians’ decision to assess driving. Our results also showed that men had more frequent assessments of dementia medications. Given that Rochon et al. [39] found women were at greater risk of having serious events following prescriptions for dementia, this finding points to the need for more careful consideration of adverse medication-related events in women with dementia. Finally, women in our study had more home care needs assessment than men. While higher rates of referral or use of home care services in women have been previously reported, women also report more unmet homecare needs [43]. Further research is needed to explore whether differential use of home care adequately meets the needs of men and women with dementia.

Our study has certain limitations. We conducted a cross-sectional analysis; thus, we can conclude on association, not causality. Our study did not have the statistical power to detect differences for each indicator separately, so our analyses on each indicator are exploratory. The present study is a secondary analysis of a previous chart review [30] and as a result, we were unable to measure certain variables of interest, such as caregiver role or direct information on participant gender. In addition, chart review data is limited to recorded information. It also may limit our ability to inform us on gender differences, as the data recorded is often biological sex.

Though research on dementia has made considerable gains, there is a critical absence of research on sex- and gender-based differences in dementia, which in turn has led to an absence of policy and guidelines designed to best answer to the specific respective needs of men and women. This study strove to fill this important knowledge gap and highlights the importance of considering sex and gender issues in the design, implementation, and evaluation of dementia strategies and interventions. While we identified areas of inequalities in the care received, it is unclear whether this represents inequities in access to care and health outcomes. Future research should focus on better understanding sex and gender-specific needs in dementia to bridge this gap and better inform dementia strategies.

## Data Availability

The datasets generated and/or analysed during the current study are not publicly available due requirements from the research ethics boards.

## Abbreviations

QAP: Quebec Alzheimer Plan
BPSD: Behavioural and Psychological Symptoms of Dementia
